# Salivary fluoride bioavailability after application of fluoride varnishes: A randomized clinical study

**DOI:** 10.1590/0103-644020256555

**Published:** 2026-01-12

**Authors:** Maria Suzi de Sousa Lopes, Lyzia Vitoria Mendes Rezende, Evanildo Canuto Paz, Glauber Campos

**Affiliations:** 1 Federal University of Piauí, Department of Restorative Dentistry, Teresina, Piauí, Brazil

**Keywords:** Fluorides, Saliva, Topical Fluorides

## Abstract

This study aimed to evaluate the bioavailability of F in saliva compartments (sediment and supernatant) after the use of fluoride varnishes (FV) with 5% sodium fluoride. In this randomized two-phase crossover clinical study, 15 participants were allocated into two groups and received application of Duraphat® and Fluorniz on the vestibular surface of the teeth. Unstimulated saliva was collected before the application of the FV, and 5 min, 15 min, 30 min, 1 h, 2 h, 4 h, 8 h, 12 h, 24 h, 48 h, and 96 h after application. For statistical analysis, a t-test was performed to compare the FV in the salivary compartments. The F in saliva at different times was compared with the baseline using analysis of variance, with Dunnett’s *post hoc* test for multiple comparisons. After application, the F concentration in the salivary compartments increased (p < 0.001) and returned to baseline 24 h after application in the Duraphat® group and 8 h after application in the Fluorniz group. When comparing the salivary sediment and supernatant, there was a difference between the FV up to 48 h after application, with higher F concentrations observed in the Duraphat® group. The availability of F was higher after the use of Duraphat® (p < 0.05). The application of Duraphat® and Fluorniz increased the F concentration in saliva, and Fluorniz presented lower F bioavailability.

## Resumo

Este estudo teve como objetivo avaliar a biodisponibilidade de flúor (F) nos compartimentos salivares (sedimento e sobrenadante) após o uso de vernizes fluoretados (VF) contendo 5% de fluoreto de sódio. Neste estudo clínico randomizado, cruzado e em duas fases, 15 participantes foram alocados em dois grupos e receberam aplicação dos vernizes Duraphat® e Fluorniz na superfície vestibular dos dentes. A saliva não estimulada foi coletada antes da aplicação dos VF e após 5 min, 15 min, 30 min, 1 h, 2 h, 4 h, 8 h, 12 h, 24 h, 48 h e 96 h da aplicação. Para a análise estatística, utilizou-se o teste t para comparar os vernizes nos compartimentos salivares. As concentrações de F na saliva em diferentes tempos foram comparadas ao valor basal por meio de análise de variância (ANOVA), com o teste pós-hoc de Dunnett para múltiplas comparações. Após a aplicação, a concentração de F nos compartimentos salivares aumentou (p < 0,001) e retornou ao valor basal 24 h após a aplicação no grupo Duraphat® e 8 h após a aplicação no grupo Fluorniz. Ao comparar o sedimento salivar e o sobrenadante, observou-se diferença entre os VFs até 48 h após a aplicação, com concentrações de F mais altas no grupo Duraphat®. A disponibilidade de F foi maior após o uso do Duraphat® (p < 0,05). A aplicação de Duraphat® e Fluorniz aumentou a concentração de F na saliva, sendo que o Fluorniz apresentou menor biodisponibilidade de F.

**Figure:**
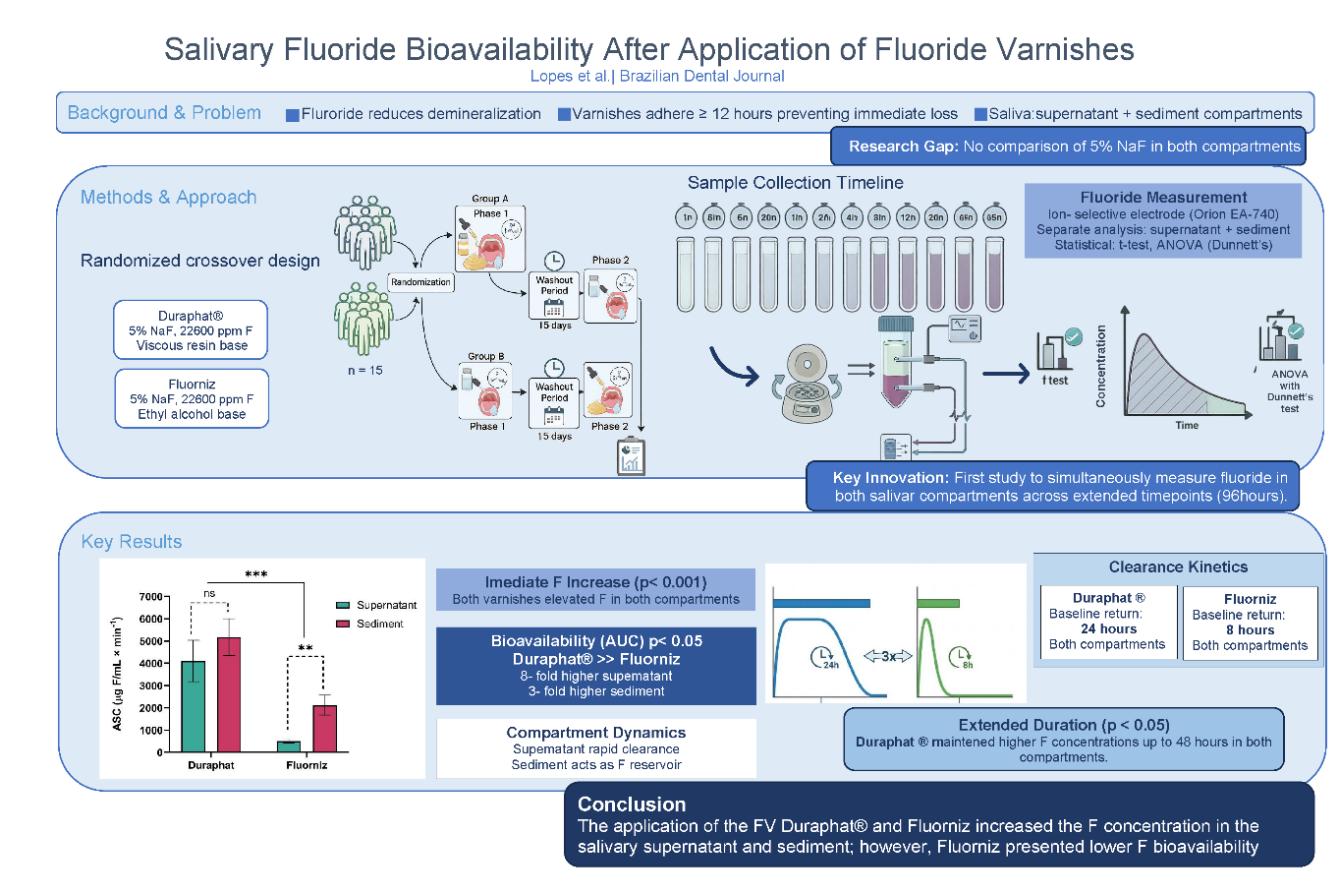

## Introduction

The use of fluorides (F) remains the most effective strategy for controlling dental caries at both individual and population levels^1^. Their action is physicochemical, reducing demineralization and enhancing remineralization of enamel and dentin^2^. For this, F must be continuously available in the oral environment at low but constant concentrations (0.02-0.05 ppm F)^3^. Oral reservoirs, such as biofilm, soft tissues, the tongue, calcium fluoride (CaF₂) formed on enamel, and saliva, maintain transient increases in fluoride levels, followed by clearance through swallowing and salivary secretion^4^. Importantly, fluoride must be free or soluble in the aqueous phase of the oral cavity (biofilm fluid or saliva) to exert its preventive effect^5^.

Fluoride bioavailability in saliva depends on the mode of administration, formulation solubility, concentration, and salivary flow^6^ ^,^ ^7^. Studies with dentifrices demonstrate that higher product concentrations yield proportionally greater fluoride levels in saliva^8^. Consequently, patients at high caries risk benefit from products that maintain elevated fluoride concentrations, such as professionally applied gels and fluoride varnishes (FV)^3^. FV were designed to extend the contact time between F and enamel by adhering to the tooth surface as a thin film for more extended periods (≥12 h), preventing immediate loss of fluoride^9^ ^,^ ^10^. Interaction with saliva leads to the formation of calcium fluoride (CaF₂) on the enamel surface, stabilized by pellicle proteins and phosphate at neutral pH. Under acidic conditions, CaF₂ dissolves, releasing F and acting as a sustained source^11^ ^,^ ^12^. Therefore, the persistence of salivary fluoride depends on product formulation, application method, and elimination rate^13^.

The use of FV is effective in reducing the prevalence and severity of caries in high-risk populations, in primary and permanent dentitions, and should be applied two to four times a year^14^. FV are widely accepted due to their ease of application, rapid setting, and high safety, with no reported short-term adverse effects^15^ ^,^ ^16^. Previous studies have mainly assessed fluoride concentrations in the salivary supernatant after FV application^12^ ^,^ ^17^ ^,^ ^18^. However, whole saliva comprises two compartments: supernatant and sediment^19^. The sediment contains organic material (bacteria, epithelial cells, proteins) and inorganic ions, including fluoride^20^ ^,^ ^21^, while the supernatant consists primarily of water and electrolytes^22^ ^,^ ^23^ ^,^ ^24^. Although bioavailable fluoride corresponds to the free ions in the supernatant, the sediment may act as a reservoir capable of binding and gradually releasing fluoride, contributing to its long-term availability^25^.

Several FV formulations are available, including those with added calcium phosphates such as casein phosphopeptide-amorphous (ACP)^26^ and tricalcium (TCP)^27^, and others containing only 5% sodium fluoride (NaF), such as Duraphat®^28^ and Fluorniz^29^. A 5% NaF FV remains the gold standard for preventing or reversing non-cavitated lesions in both dentitions^30^. Previous studies compared FV with other topical products and observed higher fluoride concentrations and longer retention in saliva after FV application^12^. Nevertheless, no studies have simultaneously compared different 5% NaF varnishes, evaluating fluoride release in both salivary compartments (supernatant and sediment) to distinguish free and bound fluoride fractions. Therefore, this study aimed to evaluate the bioavailability of fluoride in the salivary supernatant and sediment following the application of 5% NaF fluoride varnishes.

## Materials and methods

### Ethical aspects

This study was approved by the Research Ethics Committee of the Federal University of Piauí (opinion 5543818). The participants signed the Free and Informed Consent Form (FICF), in accordance with Resolution No. 466 of the National Health Council, Ministry of Health, Brasília, DF, 12/12/2012, and received verbal and written information with guidelines about the research. This study followed the ethical principles originating from the Declaration of Helsinki and adopted the CONSORT 2010 Statement as a reporting framework for randomized clinical trials. The trial was prospectively registered in the Brazilian Clinical Trials Registry (RBR-8974jb6), and no deviations or amendments occurred between the registered protocol and the final manuscript.

### Pilot study

The pilot study was conducted with six participants distinct from those included in the main trial, for the purpose of determining collection times, standardization, and procedural adjustments.

### FV and toothpaste

Two commercially available FVs were tested: Duraphat® (Colgate-Palmolive, Eschwege, Germany) and Fluorniz (SS White, Rio de Janeiro, Brazil), both containing 5% NaF or 22,600 ppm F. Condor Baby toothpaste (Condor, SC, Brazil) without F was also used in the study (Box 1).

**Box 1:**
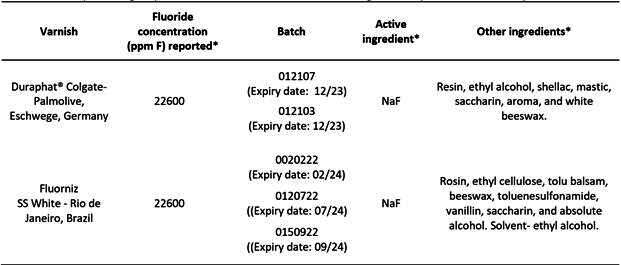
Description of groups, fluoride content of varnishes and ingredients present in the composition.

*Informed by the manufacturer. NaF = Sodium Fluoride

### Recruitment

The participants invited to participate in the study were undergraduate or graduate students in Dentistry at UFPI. After agreeing to participate in the study, they were assessed according to the inclusion criteria, such as having unrestored vestibular surfaces of the teeth, the absence of active carious lesions, and normal salivary flow. To assess salivary flow, a millimeter-sized tube was made available for unstimulated saliva collection, using the spitting technique, and the total sample obtained was assessed. If the participant managed to collect 2 mL of saliva in 2 min, they were included in the study^28^. Although the inclusion criterion of 2 mL in 2 min may be higher than the average unstimulated flow rate, it was based on pilot data confirming normal flow in our study population (mean 1.1 mL/min), consistent with reference physiological ranges^22^. In turn, participants who used medications that alter salivary flow, used F supplements, smoked, had periodontal disease, had fixed orthodontic appliances, were allergic or sensitive to the ingredients of the FV used in the study, or had received FV in the last 3 months were excluded from the study. Before the stages were carried out, the participants were seen at a clinic and assessed according to the inclusion criteria and salivary flow rate.

The sample size calculation was performed using G*Power software, version 3.1.9.7 (Heinrich Heine University, Düsseldorf, Germany). It was based on the primary outcome, fluoride concentration (µg F/mL) in the salivary supernatant, derived from pilot data comparing fluoride varnishes at 48 hours post-application, which represented the smallest difference observed between groups. An alpha error of 5% and a beta error of 10% were considered, resulting in a total sample of 15 participants and an actual statistical power of 0.92.

### Experimental design

**Figure 1:**
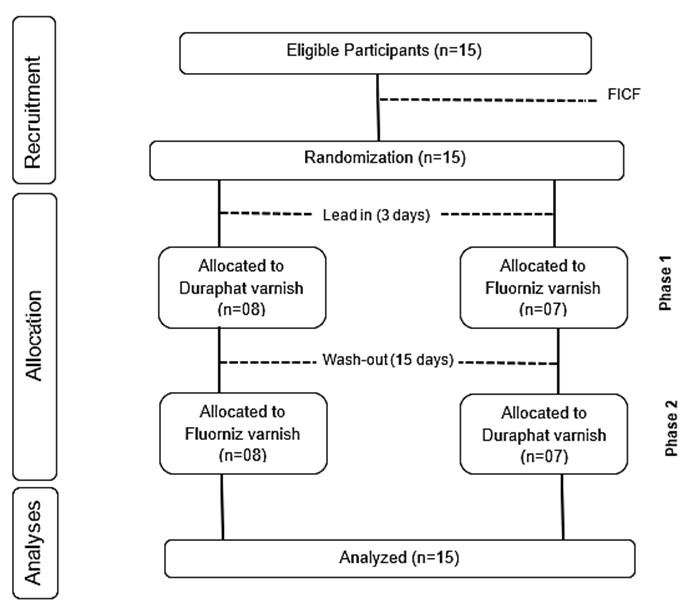
Flowchart of the experimental design of the study.

This randomized, crossover, double-blind clinical study was conducted with 15 adults living in a municipality with a fluoridated public water supply. During two experimental phases, the participants were randomly allocated into two groups and received two types of treatments: Duraphat® and Fluorniz. Group A (n=8) received Duraphat® in Phase 1 and Fluorniz in Phase 2, whereas Group B (n=7) received the reverse order. The FV was applied to the vestibular surface of the upper and lower teeth, with the exception of the third molar. The randomization of the subjects, the application of the FV, and preparation of the samples were performed by the same individual who knew the type of FV applied. However, the analyses of the saliva samples were performed by a researcher who was unaware of this information. Before the beginning of the experimental phases, the participants used non-fluoridated toothpaste for 3 days (lead-in), and between phases, they used the same toothpaste for 15 days (washout). The flowchart of the experiment is shown in Figure 1.

### Application of the FV

The FV was applied by a single professional, according to the manufacturer's instructions. The participants underwent dental prophylaxis with a pumice stone (SS White) and a Robinson brush (American Burrs, Santa Catarina, Brazil). Duraphat® is presented in a single package, and 2 mL of the product was separated for application. Fluorniz is available in two bottles, one for the varnish and one for the solvent. For the application, a mixture was obtained with the proportion of 1.5 mL of varnish and 0.5 mL of solvent, totaling 2 mL. The amount of each FV was determined with the aid of a disposable syringe. After performing relative isolation and with the teeth dry, the FV were applied to the vestibular surface of all teeth, except the third molars, using a microbrush (KG Korense, São Paulo, Brazil), using 2 mL of product. The participants were instructed to avoid hard foods, not to brush their teeth for the first 24 h, not to consume foods containing F, such as sardines and green tea, and not to eat or drink, with the exception of water, 1 h before any of the saliva collection times. Twenty-four hours after applying the products, the participants were allowed to brush their teeth with the toothbrush and fluoride-free toothpaste provided by the researcher.

The randomization sequence was generated by an independent researcher using Microsoft Excel and stored in a password-protected file. Allocation concealment was ensured using sealed, opaque, sequentially numbered envelopes, which were opened only at the time of varnish application. The independent researcher who generated the sequence was not involved in participant enrollment or intervention. The participants were blinded regarding the order of FV application and name; however, due to the uniqueness of each varnish (flavor, color, and adhesion), the participants had the potential to discriminate between the FV. However, no participant reported curiosity in discovering the FV that had been applied.

### Sample collection

Unstimulated saliva collection, using the spitting technique, was performed before the application of the FV (baseline), and then 5 min, 15 min, 30 min, 1 h, 2 h, 4 h, 8 h, 12 h, 24 h, 48 h, and 96 h after application. The participant collected the saliva in a plastic tube for a maximum of 4 min at each collection time, with an approximate quantity of 3 mL. After collection, each plastic tube was stored at -20 °C until analysis^28^.

### Determination of the salivary F concentration

A specific electrode (model 18AF-001, Analyser Instrumentação Análise, São Paulo, SP, BR) coupled to an ion analyzer (Orion EA-740) was used to measure the salivary F concentration. Aliquots (1 mL) of the thawed samples were collected and centrifuged for 3 min. Then, 0.5 mL of the salivary supernatant was buffered with 0.05 mL of TISAB III (06). The F concentration was calculated by linear regression of two calibration curves, obtained from F concentration standards. The samples collected 5 min, 15 min, 30 min, 1 h, 2 h, and 4 h after application were evaluated with the high-concentration curve using the 0.25-32 ppm F standards. The samples collected at baseline and 8, 12, 24, 48, and 96 h after application were evaluated with the low-concentration curve using the 0.0625-2.0 ppm F standards.

The salivary supernatant remaining after the first analysis was discarded, leaving only the sediment. Aliquots (0.5 mL) of TISAB II were added to the sediment and stirred for 10 s in a vortex mixer (Nova Instrumentos, Piracaba, SP, BR)^21^. The suspension was analyzed to determine the F concentration using a specific electrode (model 18AF-001, Analyser Instrumentação Análise) coupled to an ion analyzer (Orion EA-740), in a similar manner to that performed for the salivary supernatant.

### Statistical analysis

The Shapiro-Wilk test was used to determine whether the data followed a normal distribution. It revealed the non-normal distribution of the data. Thus, the data were log10 transformed and evaluated with the t-test for comparisons of the two FV at each collection time, in the salivary supernatant and sediment. The salivary F concentrations after application of the FV at the different time intervals were compared with the baseline concentration using analysis of variance with Dunnett’s *post hoc* test for multiple comparisons. The descriptive statistical data are presented as graphs and tables, and GraphPad Prism 9.51 (GraphPad, La Jolla, CA, USA) was used for analysis.

## Results

There was no difference in the salivary F concentration at baseline between the two FV (p > 0.05). Table 1 shows the F concentration in the salivary supernatant and sediment at different times after application of the FV. Immediately after the application of both FV, the F concentration in the salivary supernatant and sediment increased significantly (p < 0.001), and decreased at all other times, returning to baseline 24 h after application in the Duraphat® group, and 8 h after application in the Fluorniz group. When comparing the salivary sediment and supernatant between the groups, there was a difference between the Duraphat® and Fluorniz FV up to 48 h after application, with a higher salivary F concentration in the Duraphat® group compared with the Fluorniz group.

**Table 1:** Salivary fluoride concentration (Mean ± SD, µg F/mL) in the supernatant or salivary sediment after application of the varnishes evaluated.

Time	Supernatant		p-value *	Sediment		p -value
	Duraphat ®	Fluorniz		Duraphat ®	Fluorniz	
baseline	0.02 ± 0.01	0.03 ± 0.02	0.059	0.14 ± 0.04	0.12 ± 0.02	0.103
5 min	122.63 ± 106.66 ^α^	13.03 ± 8.82 ^α^	<0.001*	35.72 ± 17.36 ^α^	8.97 ± 7.45 ^α^	<0.001*
15 min	28.21 ± 11.61 ^α^	4.68 ± 4.95 ^α^	<0.001*	15.26 ± 8.95 ^α^	5.93 ± 4.18 ^α^	0.003*
30 min	18.50 ± 10.52 ^α^	1.90 ± 1.84 ^α^	<0.001*	9.84 ± 4.99 ^α^	4.44 ± 3.67 ^α^	0.004*
1 h	12.40 ± 8.21 ^α^	0.94 ± 0.81 ^α^	<0.001*	9.79 ± 4.13 ^α^	3.75 ± 2.92 ^α^	0.003*
2 h	7.00 ± 5.22 ^α^	0.51 ± 0.49 ^α^	<0.001*	7.85 ± 3.74 ^α^	1.79 ± 1.79 ^α^	<0.001*
4 h	2.46 ± 2.34 ^α^	0.29 ± 0.29 ^α^	<0.001*	4.65 ± 3.21 ^α^	1.17 ± 1.21 ^α^	<0.001*
8 h	1.14 ± 1.42 ^α^	0.08 ± 0.06 ^α^	<0.001*	2.23 ± 1.76 ^α^	0.58 ± 0.52 ^α^	0.001*
12 h	0.41 ± 0.65 ^α^	0.04 ± 0.03	<0.001*	0.83 ± 0.63 ^α^	0.39 ± 0.22 ^α^	0.024*
24 h	0.18 ± 0.25 ^α^	0.04 ± 0.05	0.043*	0.54 ± 0.31 ^α^	0.33 ± 0.30	0.016*
48 h	0.04 ± 0.04	0.03 ± 0.01	0.016*	0.31 ± 0.20	0.19 ± 0.13	0.002*
96 h	0.02 ± 0.01	0.02 ± 0.01	0.195	0.22 ± 0.19	0.18 ± 0.12	0.325

*indicate a statistical difference between the two varnishes at each collection time in the salivary compartments evaluated. α indicates

the observation period in which F concentrations are higher than the baseline after Dunnett's multiple comparisons test (p < 0.05)

Comparison of the salivary components of each group (Table 2) revealed that after application of Duraphat®, there was a difference in the F concentration at all times, except for 1 and 2 h (p > 0.05). The salivary F concentration was significantly higher in the supernatant compared with the sediment up to 30 min after application, and from 4 h onwards, the concentrations were higher in the salivary sediment (p < 0.05). With the use of Fluorniz, only 5 and 15 minutes after application did not present differences. The F concentration was higher in the salivary sediment (p < 0.001).

**Table 2:** Salivary fluoride concentration (Mean ± SD, µg F/mL) after application of Duraphat and Fluorniz varnishes in the salivary compartments evaluated.

Time	Duraphat ®		p-value *	Fluorniz		p -value
	Supernatant	Sediment		Supernatant	Sediment	
baseline	0.02 ± 0.01	0.14 ± 0.04	<0.001*	0.03 ± 0.02	0.12 ± 0.02	<0.001*
5 min	122.63 ± 106.66	35.72 ± 17.36	<0.001*	13.03 ± 8.82	8.97 ± 7.45	0.082
15 min	28.21 ± 11.61	15.26 ± 8.95	0.001*	4.68 ± 4.95	5.93 ± 4.18	0.077
30 min	18.50 ± 10.52	9.84 ± 4.99	0.004*	1.90 ± 1.84	4.44 ± 3.67	<0.001*
1 h	12.40 ± 8.21	9.79 ± 4.13	0.996	0.94 ± 0.81	3.75 ± 2.92	<0.001*
2 h	7.00 ± 5.22	7.85 ± 3.74	0.221	0.51 ± 0.49	1.79 ± 1.79	<0.001*
4 h	2.46 ± 2.34	4.65 ± 3.21	0.017*	0.29 ± 0.29	1.17 ± 1.21	<0.001*
8 h	1.14 ± 1.42	2.23 ± 1.76	0.012*	0.08 ± 0.06	0.58 ± 0.52	<0.001*
12 h	0.41 ± 0.65	0.83 ± 0.63	0.001*	0.04 ± 0.03	0.39 ± 0.22	<0.001*
24 h	0.18 ± 0.25	0.54 ± 0.31	<0.001*	0.04 ± 0.05	0.33 ± 0.30	<0.001*
48 h	0.04 ± 0.04	0.31 ± 0.20	<0.001*	0.03 ± 0.01	0.19 ± 0.13	<0.001*
96 h	0.02 ± 0.01	0.22 ± 0.19	<0.001*	0.02 ± 0.01	0.18 ± 0.12	<0.001*

*indicate statistical difference between salivary compartments in each varnish studied (p < 0.05).

**Figure 2:**
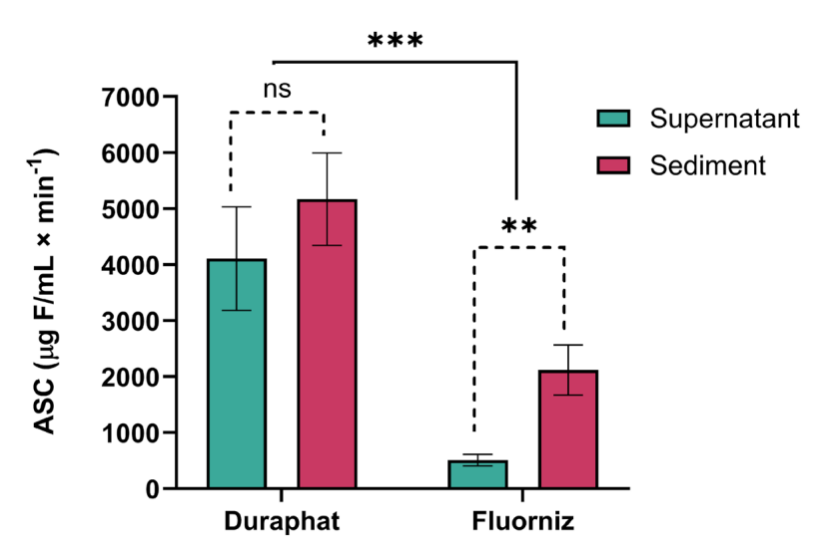
Mean area under the curve (AUC) of salivary F concentration as a function of time (μg F/mL.min ^-1^) according to treatments (n = 16). ^ns^ indicates non-significant difference (p > 0.05), **indicates statistical difference between the salivary compartments for Fluorniz varnish (p < 0.05). ***indicates statistical difference between the two varnishes for both salivary compartments (p < 0.05). The vertical bars indicate the standard deviation.

Figure 2 shows the area under the curve (AUC) of the salivary F concentration over time. The treatments differed significantly from each other, with higher F bioavailability in the salivary supernatant and sediment after the use of Duraphat® compared with Fluorniz (p < 0.05). The F concentration in the salivary supernatant and sediment after the use of Duraphat® did not present a difference (p > 0.05). However, there was a significant difference between the salivary compartments for Fluorniz, with a higher F concentration in the sediment (p < 0.05).

Table 3 shows the ratio between the average F concentration in the salivary sediment and supernatant after application of the FV. With Duraphat®, only at 5 min, 15 min, 30 min, and 1 h after application was there a higher F concentration in the supernatant compared with the sediment, while in the Fluorniz group, the F concentration was higher in the supernatant only at 5 min after application. At other times, in both groups, the ratio of the salivary compartments showed increasing behavior, even 96 h after application, identifying a higher F concentration in the salivary sediment.

**Table 3:** Proportion of mean values of fluoride concentration in the sediment to mean fluoride values in the salivary supernatant in the varnishes evaluated.

Varnish	Time											
	Baseline	5 min	15 min	30 min	1 h	2 h	4 h	8 h	12 h	24 h	48 h	96 h
Duraphat ®	8.97	0.29^*^	0.54^*^	0.53^*^	0.79^*^	1.12	1.89	1.95	2.03	3.07	6.95	9.91
Fluorniz	4.75	0.69^*^	1.27	2.33	3.98	3.49	4.02	7.15	8.69	7.36	7.29	9.77

* indicate higher fluoride concentration in the supernatant than in the sediment.

## Discussion

This clinical study is the first to evaluate two FVs with similar fluoride concentrations (5% NaF; 22,600 ppm F), Duraphat® and Fluorniz, by monitoring fluoride levels in both salivary supernatant and sediment over time. Although these varnishes share the same nominal fluoride concentration, they differ in formulation and physicochemical properties, which significantly influence fluoride bioavailability. Both products increased fluoride concentrations in saliva; however, Duraphat® maintained markedly higher levels in both compartments throughout the evaluation period. Similar differences among NaF-based varnishes have been reported previously^18^, reinforcing that formulation characteristics play a critical role in fluoride release and retention.

Previous studies have primarily examined fluoride concentrations in the salivary supernatant, neglecting the sediment fraction^12^ ^,^ ^18^. The present results confirm that the fluoride clearance pattern differs between these compartments. The more rapid decline observed in the supernatant may reflect the immediate dilution and swallowing of soluble fluoride, whereas ions bound to components of the salivary sediment are released more slowly^21^. Consequently, the sediment, in addition to the oral soft tissues, recognized reservoirs of fluoride retention^31^, appears to act as a slow-release reservoir, extending fluoride availability in the oral environment^21^.

Duraphat® demonstrated higher fluoride levels in the supernatant up to one hour after application, followed by predominance in the sediment, while Fluorniz showed a brief peak at five minutes before a faster decline. These findings indicate formulation-dependent dynamics: lower-viscosity products, such as Fluorniz, exhibit reduced adhesion to tooth surfaces and greater diffusion away from the site of application, leading to more rapid clearance and possible partial ingestion before complete fluoride release^18^. Conversely, Duraphat®, with its more viscous resin base, retains fluoride longer, favoring gradual ion diffusion and sustained salivary fluoride concentrations.

In both products, peak fluoride levels occurred five minutes after application, followed by a progressive decrease. Duraphat® maintained concentrations above baseline for up to 24 h, while Fluorniz returned to baseline within 8-12 h in the supernatant and sediment, respectively. These findings are consistent with previous studies showing that fluoride levels increase immediately after FV application but generally return to baseline within 24 h^12^ ^,^ ^18^ ^,^ ^28^ ^,^ ^32^. This period coincides with the peak formation of calcium fluoride (CaF₂) on enamel surfaces^33^, suggesting that after 24 h, most fluoride remains bound to enamel rather than freely circulating in saliva.

When comparing overall fluoride bioavailability, Duraphat® exhibited approximately eightfold higher concentrations in the supernatant and threefold higher in the sediment than Fluorniz. These differences are attributed to compositional and rheological variations. Duraphat® contains a natural resin base with a small proportion of alcohol and chitosan, enhancing viscosity and reducing wettability, which promotes prolonged fluoride retention and slower ion diffusion^34^. In contrast, Fluorniz, supplied in two separate components (varnish and solvent), contains ethyl alcohol that increases fluidity and volatility, facilitating rapid evaporation and fluoride loss^29^ ^,^ ^35^. The higher viscosity and cohesive structure of Duraphat® likely explain its superior fluoride bioavailability and clinical effectiveness.

Salivary fluoride levels are directly related to the amount of fluoride available to interact with enamel surfaces^36^. Continuous exposure to low concentrations of fluoride in saliva and biofilm fluid is critical to counteracting demineralization by promoting fluorapatite formation and facilitating remineralization through calcium and phosphate deposition^1^. Several studies have confirmed that FV maintains salivary fluoride at effective levels for extended periods, reduces lesion depth, and enhances enamel remineralization compared with other topical fluoride products^12^ ^,^ ^35^ ^,^ ^37^.

As highlighted by Marinho *et al.* 2013^14^, FV application to either primary or permanent teeth substantially reduces caries progression. This efficacy derives from the ability of varnishes to sustain contact between fluoride and enamel in a thin, adherent film for ≥12 h, minimizing loss through swallowing and acting as a long-term fluoride reservoir^9^. During this period, fluoride ions from the varnish dissolve into saliva, are absorbed by oral reservoirs, including enamel, plaque, and soft tissues, and are gradually re-released, combining with calcium to form CaF₂. These reservoirs dissolve slowly, releasing fluoride ions that provide ongoing protection against caries^38^.

The ratio of fluoride concentration between sediment and supernatant increased over time for both varnishes. In Duraphat®, fluoride levels were initially higher in the supernatant (ratio < 1) up to 1 h post-application, shifting to predominance in the sediment thereafter (ratio > 1). For Fluorniz, this shift occurred after only 5 min. These dynamics further support the role of salivary sediment as a retention compartment and the superior persistence of fluoride derived from Duraphat®. Santos *et al.* 2009^35^ found similar results in vitro, showing that Duraphat® reduced lesion depth by 58%, compared with 35% for Fluorniz, reinforcing the greater potential of more viscous formulations to maintain fluoride in the oral cavity and enhance anticariogenic effects.

The dual-compartment analysis applied in this study reflects the pharmacokinetic behavior of fluoride in a semi-homeostatic system such as saliva, which is influenced by interindividual differences in flow, biochemical composition, and microbial activity^6^. We acknowledge that variation in sample collection times, particularly those performed at night when salivary flow is reduced^39^, may have affected fluoride concentration measurements. Nonetheless, this limitation does not alter the overall trends observed. Finally, although cost was not the primary focus of this study, our findings have clinical implications for product selection in public health programs. Despite its lower price, Fluorniz exhibited substantially lower fluoride bioavailability than Duraphat®, emphasizing that formulation characteristics and not only cost should guide the choice of fluoride varnish in preventive protocols^40^.

## Conclusion

The application of the FV Duraphat® and Fluorniz increased the F concentration in the salivary supernatant and sediment; however, Fluorniz presented lower F bioavailability. Therefore, the F concentration in the product is not the only factor influencing salivary fluoride bioavailability. This study introduces a novel approach by evaluating fluoride bioavailability in the salivary sediment, highlighting its role as a potential reservoir for slow fluoride release, contributing to prolonged fluoride presence in the oral cavity.
